# Functional Dissection of the Dominant Role of CD55 in Protecting Vesicular Stomatitis Virus against Complement-Mediated Neutralization

**DOI:** 10.3390/v13030373

**Published:** 2021-02-26

**Authors:** Nisha Asok Kumar, Sreenath Muraleedharan Suma, Umerali Kunnakkadan, Joydeep Nag, Reshma Koolaparambil Mukesh, Douglas S. Lyles, John Bernet Johnson

**Affiliations:** 1 Pathogen Biology, Virology, Rajiv Gandhi Center for Biotechnology, Thiruvananthapuram, Kerala 695014, India; nishaasok@rgcb.res.in (N.A.K.); sreenathms9@gmail.com (S.M.S.); umeralik@rgcb.res.in (U.K.); joydeep@rgcb.res.in (J.N.); reshmakm@rgcb.res.in (R.K.M.); 2 Manipal Academy of Higher Education, Manipal, Karnataka 576104, India; 3 Department of Biotechnology, University of Kerala, Thiruvananthapuram, Kerala 695581, India; 4 Department of Biochemistry, Wake Forest School of Medicine, Winston-Salem, NC 27101, USA; dlyles@wakehealth.edu

**Keywords:** vesicular stomatitis virus, complement, virus neutralization, viral resistance, membrane cofactor protein (CD46), decay accelerating factor (CD55)

## Abstract

The human complement system is an important part of the innate immune system. Its effector pathways largely mediate virus neutralization. Vesicular stomatitis virus (VSV) activates the classical pathway of the complement, leading to virus neutralization by lysis. Two host-derived membrane-associated regulators of complement activation (RCA), CD55 and CD46, which are incorporated into the VSV envelope during egress, confer protection by delaying/resisting complement-mediated neutralization. We showed previously that CD55 is more effective than CD46 in the inhibition of neutralization. In this study, we identified that, at the protein level, VSV infection resulted in the down-regulation of CD46 but not CD55. The mRNA of both the RCAs was significantly down-regulated by VSV, but it was delayed in the case of CD55. The immunoblot analysis of the levels of RCAs in the progeny virion harvested at three specific time intervals, points to an equal ratio of its distribution relative to viral proteins. Besides reconfirming the dominant role of CD55 over CD46 in shielding VSV from complement, our results also highlight the importance of the subtle modulation in the expression pattern of RCAs in a system naturally expressing them.

## 1. Introduction

Innate immune barriers, including the complement system, act as the front line defense against viral infections. The triggering of one or more of the three pathways of complement activation (classical, alternative, or the MBL/lectin pathway) by viruses can potentiate a cascade of reactions supporting virus neutralization and clearance. Complement component C3 acts as a common converging point, and the cleavage product C3b is a key component of activation pathway enzymes, i.e., the C3 and C5 convertases. The activation and amplification of complement pathways can result in the assembly of the lytic complex called the membrane attack complex (MAC) [1,2] on the surface of viruses and infected cells, leading to lysis [3]. Besides the MAC mediated lysis of pathogens or infected cells, the active mechanisms of pathogen clearance by complement include: (a) the opsonization of pathogens for phagocytosis, (b) the production of anaphylotoxins (e.g., C5a, C3a), (c) aggregation, and (d) the activation and regulation of T and B cell responses [3,4,5].

Vesicular stomatitis virus (VSV), a prototypic member of the family Rhabdoviridae, has been shown to be sensitive to complement. It is known to activate the classical pathway of complement in an antibody-dependent manner, with natural IgM contributing significantly to the complement-mediated neutralization of VSV [6,7,8,9]. However, there are marked differences in the complement neutralization of VSV generated in cell lines of different species, independent of the antibody, facilitated via the direct engagement of C1 to the envelope glycoprotein (G) of VSV [10]. The upstream complement components of the classical pathway, including C1, C2, C4 and C3, were found to be essential for VSV neutralization [6,7,8]. Complement activation by VSV results in the deposition of opsonins such as C3b on the virion, promoting an initial aggregation, followed by lysis [11]. It has been demonstrated that the involvement of complement components (C3, C4) and complement receptors (CR2, CR3 and CR4) in the resident macrophages in the marginal zones of the secondary lymphoid organs is pivotal in the engagement of B-cell specific responses during VSV infection [12].

The complement system is tightly regulated by a set of regulatory proteins referred to as ‘regulators of complement activation’ (RCA) or ‘complement regulatory proteins’ (CRPs/Cregs). They function to limit the apparent ‘bystander effects’ exerted by complement activation on healthy host tissues [13,14,15]. The RCAs are either soluble, functioning in fluid phase (e.g., C4b-binding protein (C4BP), factor H (FH)), or are membrane bound, acting on the cell surface (e.g., CD55 (decay accelerating factor, DAF), CD46 (MCP or membrane cofactor protein) [16], and CD35 (complement receptor I, CR1)) [14,17]. CD55 is a GPI-anchored type I glycoprotein [18] which is widely expressed on cells of multiple origins, although its expression differs from one cell type to another. CD55 exerts its regulatory function, decay accelerating activity (DAA), by binding to the C3 and C5 convertases of both the classical and the alternative pathway, and rapidly dissociating them into their constituents [19,20]. CD46, in contrast, is a type I trans-membrane protein which is ubiquitously expressed in all nucleated cells. It functions as a cofactor for the factor I (fI)-mediated inactivation of C3b, deposited on the cell surface into its inactive form, iC3b [16,21]. Viruses have coevolved with their hosts, and have adopted ingenious evasive strategies against complement, thus evading its neutralizing effects. One such strategy adopted by enveloped viruses includes the hijacking of the host membrane-bound RCAs. For instance, CD55 and CD46 are incorporated into the envelopes of viruses including parainfluenza virus 5 (PIV5), mumps virus (MuV), and vesicular stomatitis virus (VSV) [11,22,23].

In our previous study, we documented the association of two host complement regulatory proteins, CD55 and CD46, in vesicular stomatitis virions budding from Chinese Hamster Ovary (CHO) cells stably transfected with CD55 and CD46, either individually or together [11]. The recruitment of host proteins does not always translate to biological function [24], and thus may not contribute productively to the virus life cycle. However, we showed that both the CD46 and CD55 associated with VSV were functional, with CD46 acting as a cofactor for the factor I mediated cleavage of C3b into iC3b, while CD55 possessed decay-accelerating activity against both classical and alternative pathway convertases [11]. Furthermore, our studies and others have also validated the protective effect that CD55 has over CD46 in conferring resistance to complement-mediated neutralization [25,26].

The transcriptional and translational inhibition of expression of host cellular proteins by VSV has been well established [27,28]. This raises the question of the potential impact of this inhibition on the cellular levels of RCAs, CD55, and CD46 upon VSV infection. Most of the previous studies have focused only on the recruitment/association of CD46 and CD55 by VSV, and the functional and protective role of these RCAs. Therefore the goal of this study was to determine the effect of VSV infection on CD46 and CD55 expression in infected cells, their incorporation into the virus envelope, and its role in protection against complement. Our results show that VSV infection causes the transcriptional shutdown of CD46 and CD55 mRNA expression, like many other host proteins; however, it also causes an effective delay in the case of CD55. In contrast to mRNA, the surface and total protein levels of CD55 remained unaltered even at later time points, while a decline in CD46 levels was evident as early as 12 hpi. Although the relative half-life of the physiological levels of CD55 compared to CD46 was not significantly different, the overall levels were. Our findings thus support a dominant role for CD55 over CD46 in protecting VSV from complement-dependent neutralization.

## 2. Materials and Methods

### 2.1. Statement on Biosafety Clearance

The rwt strain of Indiana vesiculovirus was rescued using the established reverse genetics approach. The approval pertaining to use of VSV for in vitro experiments was obtained from the Institutional Biosafety Committee, Rajiv Gandhi Centre for Biotechnology, Thiruvananthapuram, India.

### 2.2. Cell Lines and Virus

The HeLa and A549 cells were maintained in Dulbecco’s modified Eagle medium (DMEM), and the Vero cells were maintained in Minimum Essential Medium (MEM) supplemented with 10% heat inactivated fetal bovine serum (FBS) and 1X Penicillin-Streptomycin-Glutamine (PSG) (Gibco) at 37 °C, 70% humidity, and 5% CO_2_ in a CO_2_ incubator. The wild type VSV (Indiana vesiculovirus, rwt strain) was prepared by culturing the virus in HeLa cells maintained in DMEM containing 2% FBS and 1X PSG for 12–18 h [29]. The culture supernatant containing the virus was clarified by centrifugation at 4000 rpm for 10 min at 4 °C, and was supplemented with 0.75% BSA, made into aliquots and frozen at −80 °C. The viral titer was determined by a plaque assay on the Vero cells.

### 2.3. Antibodies

The rabbit and mouse monoclonal antibodies against CD55 and CD46 used in flow cytometry experiments, and the recombinant CD55 and CD46 were sourced from Sino Biological Inc. (Beijing, China). The polyclonal anti-CD55 (sc-7153) and anti-CD46 (sc-9098), and monoclonal anti-β-actin (A1978) antibodies used in all of the Western blotting experiments were obtained from Santa Cruz Biotechnology (Santa Cruz, CA, USA) and Sigma (St. Louis, MO, USA), respectively. The monoclonal antibodies against VSV-G (8G5F11) and VSV-M (23H12) were kindly provided by Prof. Douglas S. Lyles.

### 2.4. Western Blotting

HeLa cells grown to confluency (90–95%) were either mock-infected or infected with wt-VSV at a Multiplicity of Infection (MOI) of 10 for 1 h. The cells were washed once with PBS, and the media were replaced with DMEM containing 2% FBS and 1XPSG. The cells (attached as well as floating) from the VSV-infected and mock-infected dishes were lysed with 1% SDS at specific time points from 0 to 24 h. The lysates were boiled at 100 °C for 5 min, and then sonicated at an amplitude of 65% for 5 min (Branson SFX550 Sonifier, Danbury, CT, USA). The protein concentration of the lysates was then estimated using a BCA reagent (Thermo-Pierce, Chicago, IL, USA), as per the manufacturer’s protocol. In total, 30 μg cell lysate protein was separated on a 10% SDS-PAGE gel and transferred to a nitrocellulose membrane. The blots were probed with specific primary antibodies against human CD46, CD55, VSV M protein, and human β-actin, and their respective HRP-conjugated secondary antibodies. The blots were developed using SuperSignal West Pico chemiluminescent substrate (Thermo-Pierce, Chicago, IL, USA), and were exposed to X-ray film. The blots were stripped in 0.2 M glycine HCl pH 2.3 and re-probed. Three independent infection experiments and Western blots were performed in order to verify the observation.

### 2.5. Taqman Real-Time PCR

The total RNA was extracted from the mock- and VSV-infected HeLa cells with Trizol reagent (Thermo Scientific, Chicaco, IL, USA). Approximately 2000 ng of RNA was converted to cDNA using a high capacity cDNA synthesis kit (Applied Biosystems, Foster City, CA, USA), and was then diluted 1:20 in nuclease-free water. The real time PCR was set up in a total volume of 20 μL using 10 μL fast advanced master mix, 4 μL cDNA (from 1:20 diluted), 1 μM gene-specific primer, and 18s rRNA-specific primer and nuclease free water. FAM-labelled gene specific primers and VIC-labeled 18s rRNA primers (Table 1) were procured from Applied biosystems. The reaction was carried out in a 96-well plate in the Applied Biosystems™ QuantStudio™ 7 Flex Real-Time PCR System. Each sample was tested in triplicates from three independent experiments. The comparative Ct method was used for the data analysis.

### 2.6. Cycloheximide (CHX) Chase Assay

The HeLa cells were treated with 500 μg/mL cycloheximide (Sigma-Aldrich, St. Louis, MO, USA) and incubated at 37 °C for the indicated time points. The lysates were isolated and their protein concentration was assessed by BCA assay (Thermo Scientific, Chicaco, IL, USA). In total, 50 μg of the lysates were subjected to immunoblotting in order to compare the stability of CD55 and CD46. As a loading control, the samples were probed with anti-β actin antibody. P53 was probed as the positive control. The densitometry analysis of the band intensities was carried out using Image J software, and was plotted in GraphPad Prism 6 software (San Diego, CA, USA).

### 2.7. Flow Cytometry

The HeLa cells were either mock infected or infected with wt-VSV at an MOI of 10, and were incubated for 1, 6, 12, 18 and 24 h. At the end of the indicated time points, the cells were washed with ice cold FACS wash buffer (1X phosphate-buffered saline (PBS), pH 7.4, containing 0.1% sodium azide) and harvested. Both the floating and harvested cells were, at specific time points, subjected to centrifugation (800× *g* for 4 min at 4 °C), and were pooled. The cells were then washed and transferred to a 96-well U-bottom plate. The cells were then blocked with 2% BSA in PBS for 30 min and washed twice with FACS buffer (1X PBS pH 7.4, 1% sodium azide, 1% BSA). The cells were incubated with 1.0 μg anti-human CD55 or 1.5 μg anti-human CD46 and anti-VSV-G antibody (8G5F11, 1:2000) in FACS buffer. After the incubation, the cells were washed and counter stained with AF488 chicken anti-mouse (1:4000), AF633 goat anti-mouse (1:100) and AF633 goat anti-rabbit (1:100) secondary antibodies (Molecular probes, Eugene, CA, USA) in FACS buffer against anti-VSV-G, CD46, and CD55 respectively. The cells were then fixed with 2% PFA in PBS for 3 min, and washed and analyzed on a BD FACS ARIA II flow cytometer (BD Biosciences, San Jose, CA, USA). An analysis was carried out on 20,000 cells per sample using BD FACSDiva^TM^ software.

### 2.8. ELISA

A VSV-specific ELISA was carried out in order to estimate the concentration of VSV in the sucrose-gradient purified preparations from the following time periods: 6–12, 12–18 and 18–24 h post infection. Based on a pre-standardization, the highest concentrations for the virus samples were set to be 4 μg for the 6–12 and 12–18 hpi samples, and 10 μg for the 18–24 hpi sample, and were followed by two fold serial dilutions in PBS. MaxiSorp (Nunc) 96-well plates were coated with the diluted virus samples (50 µL/well) and incubated overnight at 4 °C. The wells were blocked with 2% skimmed milk prepared in PBS (10 mM NaPO_4_, 150 mM NaCl pH 7.4) for 2 h at 37 °C, and were washed and incubated with mouse monoclonal anti-VSV G antibody (8G5F11) at a dilution of 1:2500 for 1h at room temperature. The bound primary antibodies were detected with goat-anti mouse HRP secondary antibody (1:5000 in PBS). After incubating for 1h at room temperature, the wells were washed, and 100 μL/well 1 mM ABTS solution (2,2′-azino-bis(3-ethylbenzthiazoline-6-sulfonic acid) (Sigma-Aldrich) in 0.1 M sodium citrate buffer, pH 4.2, containing 0.003% H_2_O_2_) was added and incubated for 20 min in the dark, and the absorbance was measured at 405 nm using Tecan Spark 10 M (Tecan Trading AG, Mannedorf, Switzerland).

### 2.9. Purification of VSV and Western Blotting

Monolayers of HeLa cells grown in five T_225_ culture flasks were infected with wt-VSV at an MOI of 1.0 for 1 h at 37 °C for the virus adsorption. After 1 h, the flasks were washed and supplemented with virus growth media (DMEM containing 2% FBS). The culture supernatant was collected at every 6 h interval and replaced with fresh growth media. The culture supernatants collected at 6, 12, 18 and 24 h post infection were clarified (3000× *g*, 4 °C for 10 min in a 5418R benchtop centrifuge (Eppendorf, Hamburg, Germany)) in order to remove any cellular debris. The clarified supernatant was subjected to ultracentrifugation (151,000× *g* for 1 h at 4 °C in a SW 41Ti rotor (Beckman Coulter Optima xl-100k, Carlsbad, CA, USA)). The virus-containing pellet was reconstituted in NTE buffer (100 mM NaCl, 10 mM Tris-HCl (pH 7.4), 1 mM EDTA) and layered over a 15% and 60% (wt/vol) sucrose cushion in NTE buffer. The virus was further concentrated by subjecting it to ultracentrifugation at 151,000× *g*, 4 °C, overnight in a Beckman SW41 rotor. The virus bands were collected and diluted in ice cold NTE, and were pelleted (151,000× *g* at 4 °C for 1 h) using the same rotor. The pellets thus obtained were re-suspended in plain DMEM containing 1 × protease inhibitor cocktail (Roche Diagnostics, Mannheim, Germany), aliquoted, snap frozen in liquid nitrogen, and stored at −80 °C. In total, 5 μg of purified virus was separated on a 10% gel and subjected to Western blot analysis in order to analyze the relative incorporation of complement regulatory proteins CD55 and CD46. The densitometry analysis of the band intensities in the immunoblots was performed using Image Lab software (BioRad, Irvine, CA, USA).

### 2.10. Virus Neutralization Assay

Complement-dependent neutralization assays were carried out as described previously, with slight modifications [11]. In total, 3 × 10^8^ pfu of VSV harvested from HeLa and A549 cells at specific time intervals (0–6, 6–12, 12–18 and 18–24 h) were incubated with PBS or Normal Human Serum (NHS) (Complement Technologies, Tyler, TX, USA) at a dilution of 1:1. The virus incubated with PBS only served as the control. After incubation at 37 °C for 30 min, the viral titers were determined by a plaque assay on a monolayer of Vero cells. The reduction in the number of plaques with respect to the input virus represented the virus neutralization. The individual data points represent an average and standard deviation of three independent experiments. Student’s t-test was used for the statistical analysis, and *p* ≤ 0.05 was considered as significant.

## 3. Results

### 3.1. Differential Expression of Complement Regulatory Proteins CD55 and CD46 in VSV-Infected Cells

**Figure 1:**
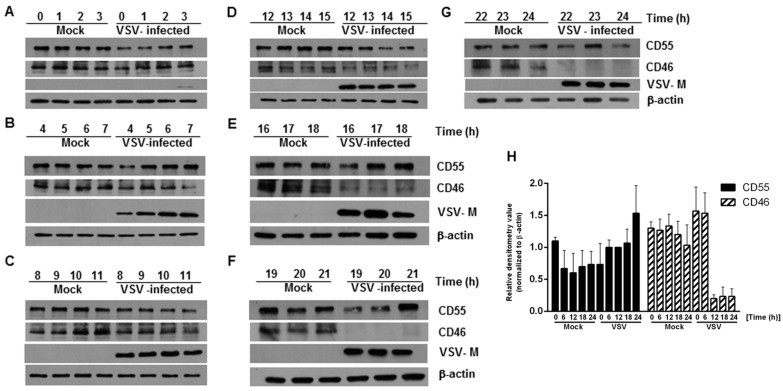
Levels of complement-regulatory protein CD55 remain unaltered, but CD46 levels decline in HeLa cells infected with vesicular stomatitis virus. (**A**–**G**) Whole cell lysates collected from mock- and VSV-infected HeLa cells at the indicated time points were subjected to immunoblotting to determine the expression of CD46 and CD55. Samples at the time point 0–3 h (**A**), 4–7 h (**B**), 8–11 h (**C**), 12–15 h (**D**), 16–18 h (**E**), 19–21 h (**F**) and 22–24 h (**G**), respectively. Anti-CD55 and CD46 antibodies were used to detect the levels of the corresponding proteins in the lysate at different time points. Virus infectivity was detected using a VSV anti-M antibody while actin served as the loading control. The levels of CD55 were maintained at all of the time points tested compared to the mock; however, the levels of CD46 declined significantly, starting from 15 h onwards (note the decreasing levels of CD46 in **D**,**E**, and the complete absence in **F**,**G**). The entire panel of blots is representative of three independent experiments. (**H**) The densitometry analysis of the CD55 and CD46 protein expression, normalized against the loading control. The data represents the mean + SEM of the three independent experiments.

In order to determine whether there are changes in the expression of CD46 and CD55 in infected cells with respect to time, HeLa cells were infected with VSV at an MOI of 10, and whole cell lysates were collected at every one hour interval up to 24 h. An immunoblot analysis was carried out on the cell lysates, and the levels of CD46 and CD55 in the infected cell lysates were compared against those of the lysates from the mock-infected cells. The immunoblot analysis of the VSV M protein expression was included as a marker of the viral protein expression. At all of the time points tested, the levels of CD55 in the VSV-infected cells were comparable to those of the mock-infected cells lysates, even up to the 24 h time point (Figure 1 top panel in A to G; Figure 1H). In contrast, the levels of CD46 in the VSV-infected cells were sustained until the 14th hour, with a gradual decline after the 15 h time point (Figure 1, second panel from the top in A to G; Figure 1H). The levels of CD46 in the infected cell lysates between 19 and 24 h post infection (hpi) were below the minimum level of detection (Figure 1, second panel from top F and G). Detectable levels of M protein were observed beginning 3 hpi (Figure 1, Second panel from bottom A to G). It should be pointed out here that it has been established that multiple isoforms of CD46 and CD55 exist in humans [30,31,32]. This accounts for the differential shift and the multiple bands observed in the immunoblots. Functionally, marked differences exist between the isoforms; for example, among the four isoforms in CD46 (BC1, BC2, C1 and C2) the BC forms have larger O-glycosylation, and were more efficient than the C forms in binding to and mediating the cleavage of the cell surface bound C4b [30]. The glycosylation on these proteins is also important functionally, with the N-glycans on the CCP-2 and CCP-4 modules of CD46 being highly essential to prevent cytolysis [33]. These data suggest that the modulation of host gene expression by VSV has a greater effect on the expression of CD46 than CD55.

### 3.2. The Cell Surface Expression of CD55 Is Greater Than That of CD46 on VSV-Infected Cells

**Figure 2:**
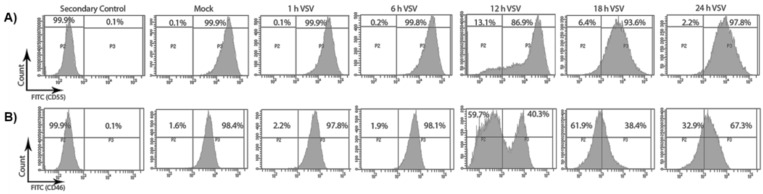
VSV infection causes a decline in the surface expression of CD46 and not CD55. HeLa cells were infected with VSV (10 MOI) for the specified time points. The surface distribution of CD55 and CD46 was determined by staining the mock- and VSV-infected cells with anti-CD55 and CD46 primary antibodies, and by counter staining with AF488-labelled secondary antibody. The two panels, **A** and **B**, denote the histogram representing the fluorescence intensity on the *x*-axis and the cell count on the *y*-axis. The infection of the HeLa cells with VSV did not alter the surface level expression of CD55 (**A**) even until 24 h; however, a drastic reduction in the surface expression of CD46 (**B**) could be evidenced 6 h post infection.

As VSV infection leads to the overall down-regulation of CD46 and not CD55, it was also important to determine whether this perturbation was reflected in the distribution of these RCAs on the cell surface. Flow cytometry was used to determine the distribution of CD46 and CD55 on the surface of the VSV-infected cells. HeLa cells were infected with VSV for 1, 6, 12, 18 and 24 h. The cell surface expression of CD46 and CD55 upon VSV infection was assessed by pooling both the attached and the floating cells, and probing with primary antibodies specific for CD55 and CD46. The fluorescence intensity of the FITC-positive population, representing the RCAs, is shown in representative histograms in Figure 2.

In the mock-treated samples, the percentages of cells expressing high levels of CD55 and CD46 were found to be 99.9% and 98.4%, respectively (Figure 2A,B, mock). In the infected samples, the percent CD55- and CD46-positive cells remained unchanged up to 6 hpi. Interestingly, by 12 hpi, a significant decline in the surface distribution of CD46 was observed. Approximately 60% of the total population at 12 hpi expressed low CD46 compared to a population of ~2% at 6hpi. Although a slight increase in the CD46-high population was observed at the 24 h time point, the expression corresponding to pre-infection levels was not attained. In contrast, the surface expression levels of CD55 remained similar to the levels in the mock-infected cells at all of the time points analyzed, except at the 12 h time point, when only ~87% of the cells were CD55-high compared to the mock levels of 99.9%. Thus, the CD55 on the cell surface was maintained even until the 24 h time point, while CD46 continued to decline after the 6 h time point.

### 3.3. VSV Down-Regulates the Expression of CD46 and CD55 mRNAs

It is already known that VSV inhibits the transcription of host mRNAs and the translation of host proteins [27,28]. This raises the question of whether the differences observed in the protein levels of CD46 versus CD55 during VSV infection corresponded to their transcript levels. In order to investigate the mRNA expression changes, HeLa cells were infected with VSV, and the total RNA was isolated by the Trizol method at 6, 12, 18 and 24 h. The cDNAs synthesized from the mock- and VSV-infected cells were subjected to quantitative real time PCR using Taqman gene expression assays.

**Figure 3:**
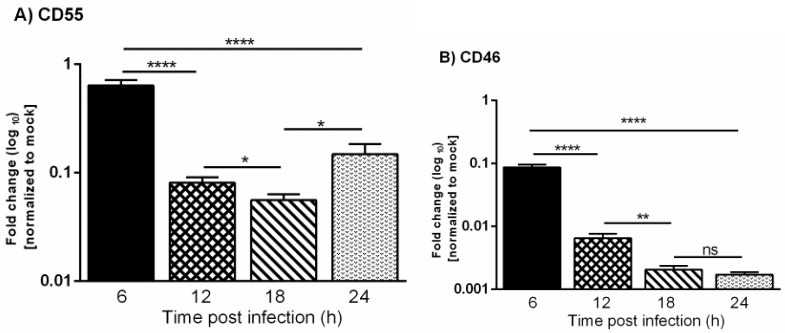
Vesicular stomatitis virus infection leads to down-regulation of CD55 and CD46 transcripts. A comparative analysis of the relative levels of CD55 (**A**) and CD46 (**B**) mRNA in VSV and mock-infected HeLa cells at 6, 12, 18 and 24 h was carried out by RT-qPCR. The total RNA isolated from the mock- and VSV-infected cells was converted to cDNA. Equal concentrations of cDNA from all of the samples were used to analyze the gene expression at various time points post VSV-infection using Taqman gene expression assays. The fold change was calculated by the comparative Ct method (2^- ddCt). The statistical significance was calculated using Students *t*-test, with * *p* ≤ 0.01; ** *p* ≤ 0.001; **** *p* ≤ 0.0001, and ns = non-significance.

The expression levels of CD46 and CD55 mRNAs in the mock and infected cells were normalized to the corresponding 18s rRNA. The fold change in the gene expression in the infected cells was calculated with respect to the mock-infected cells. The levels of CD46 transcript in the VSV-infected cells were found to be significantly lower when compared to the mock-infected cells starting from the 6 h time point, and this continued to decline rapidly as time progressed from 12 to 24 hpi (Figure 3). The levels of the CD55 transcript were also down-regulated at later times postinfection, but the transcriptional down-regulation was delayed when compared to CD46. Taken together, these results suggest that the slower decline in levels of CD55 mRNA over CD46 contributed to the sustained levels of CD55 in the protein level.

### 3.4. CD55 Is More Stable Than CD46 in HeLa Cells

**Figure 4:**
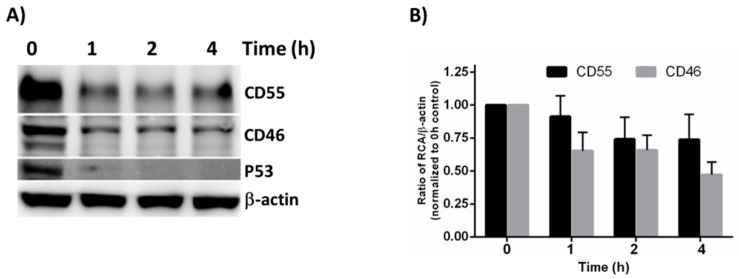
Cycloheximide chase assay to measure protein stability. (**A**) The HeLa cells were treated with Cycloheximide for the indicated times, and the whole cell lysate was subjected to immunoblotting in order to determine the stability of CD55 and CD46. β-actin was used as the loading control, and P53 served as the positive control. (**B**) Quantification of the immunoblot results by Image J software. The result represented is the average + SEM of four independent experiments obtained by normalizing the band intensity of the RCA against b-actin. The statistical significance was calculated using Student’s *t*-test.

The observation that the CD55 mRNA levels declined at later times post-infection whereas the protein levels did not suggests that differences in turnover rate contribute to the greater expression of CD55 compared to CD46. The overall turnover rate of CD55 and CD46 was determined using a cycloheximide (CHX) chase assay. The cells were treated with CHX, a known inhibitor of protein translation, for 1, 2 and 4 h, and the lysates were subjected to immunoblotting in order to determine their stability. P53 acted as the positive control, and β-actin acted as the loading control. A substantial reduction in P53 levels was observed as early as 1h (Figure 4A).

Compared to the untreated controls, the CD46 and CD55 levels were found to be reduced as early as 1 h post CHX treatment. Even though the CD55 levels appeared to be more stable than CD46 at all of the time points tested, the densitometry analysis revealed that the difference between CD55 and CD46 upon CHX treatment was insignificant (Figure 4B). Although a significant difference does not exist between the half-life of CD46 and CD55 at the time points tested, the overall higher concentration of CD55 offers a greater advantage to VSV in its recruitment, and in further imparting to it a survival advantage against complement-mediated neutralization.

### 3.5. CD46 and CD55 Incorporation into the VSV Envelope Is Independent of the Time Period of Virus Egress

**Figure 5:**
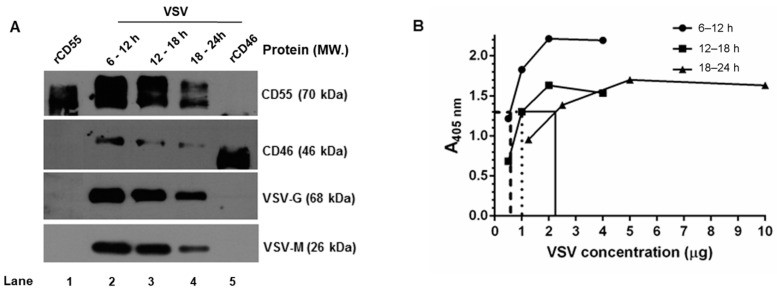
Complement regulator CD55 is found in greater abundance than CD46 on VSV from HeLa cells. (**A**) Equal concentrations of protein in the purified virus (5 μg) were separated by SDS-PAGE and subjected to Western blotting. The proteins that have been probed are indicated in the right, and their corresponding molecular weights are in parentheses. (**B**) An ELISA specific to VSV was performed by coating wells with serially-diluted gradient-purified VSV. The adsorbed virus particles were detected using an anti-VSV-G antibody. Variability in the absorbance was observed even at similar concentration of viruses purified at varying time intervals. Across the samples, an absorbance of ~1.3 was found to be common; this is indicated by the lines drawn against the optical density. (*y*-axis) to the corresponding concentrations (*x*-axis).

Despite the marked decline in both CD46 and CD55 mRNA in the VSV-infected cells, the steady maintenance in the CD55 protein levels compared to CD46 prompted the investigation of the time dependent incorporation of the RCAs in the progeny virus. The VSV released from HeLa cells was harvested at select time intervals (6–12 h, 12–18 h and 18–24 h) and purified by sucrose-gradient ultracentrifugation. The levels of CD46, CD55, G and M proteins in the gradient-purified virus were determined by immunoblotting. The analysis was carried out by loading an equal concentration of the virus based on the total protein concentration estimated by the BCA. At all of the time points tested, the incorporation of CD55 was higher than that of CD46. The maximal incorporation of the RCAs was observed in the 6–12 h sample, followed by that of the 12–18 and 18–24 h samples (Figure 5A). The band intensities of CD46 and CD55 corresponded with the viral protein concentration, which was also found to be more abundant in the 6–12 and 12–18 h samples, and comparatively less in the 18–24 h samples.

It should be pointed out here that, based on BCA, equal concentrations of the sample were taken for the analysis, yet from the signal strength of the G and M proteins in the immunoblots, it is quite evident that the band intensities corresponding to the viral proteins (G and M) are unequal. A VSV-G–specific ELISA was carried out with these purified viruses in order to account for this variability. The viruses harvested at 6–12, 12–18, and 18–24 h were first allowed to adsorb at the indicated protein concentrations, as described in the materials and methods, and were detected using a monoclonal antibody against VSV-G protein (Figure 5B). A common point of absorbance across all of the three samples was found to be 1.3, and the corresponding virus concentrations were 0.5 μg, 1.0 μg, and 2.5 μg, respectively. This marked variation corresponded with the immunoblotting results, and suggested that differences in viral proteins including G can exist in preparations across the time range [34].

Furthermore, a densitometry analysis of the band intensities of CD55 and CD46 in comparison to that of the VSV-G and M proteins (Figure 5A) was carried out in order to identify the relative levels of incorporation of the RCAs with respect to the viral proteins. Despite the visual differences observed in the immunoblots, upon normalization against G or the M proteins, the relative levels of CD46 or CD55 in the virion were found to be close across the time intervals (Table 2). However, significant differences were observed between the RCAs, with the levels of CD55 being greater than those of CD46 at all of the time points. These results suggest that RCA incorporation in VSV is independent of the time of egress for both CD46 and CD55, with the latter being more predominantly incorporated than CD46.

### 3.6. Cell Type Differences in the Protection of VSV against Complement

**Figure 6:**
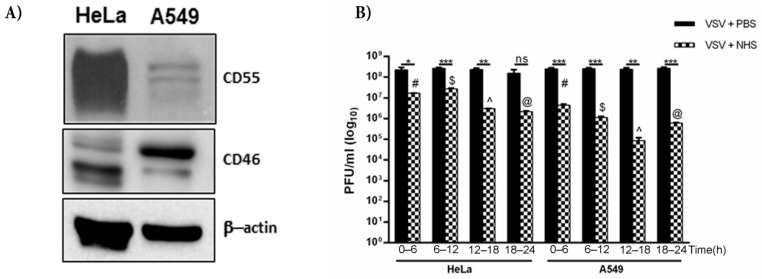
CD55 confers greater resistance to VSV against complement compared to CD46. (**A**) Western blot depicting the level of expression of CD55 and CD46 in HeLa and A549 cells; β-actin served as the equal loading control. (**B**) The effect of NHS in neutralizing VSV grown in HeLa and A549 cells was assessed by a plaque reduction assay. The virus harvested at the indicated time intervals was incubated either with NHS or PBS (black bars). At all of the time points tested, the HeLa-grown viruses showed marked resistance to complement-mediated neutralization. The degree of neutralization of A549-grown VSV known to harbor less CD55 was significantly higher than that of HeLa-grown VSV at 12–18 h (*p* < 0.0001). The symbols in the graph represent * *p* < 0.05; ** *p* < 0.005; *** *p* < 0.0005). The additional symbols represent the comparison of significance between A549- and HeLa-grown VSV treated with NHS at the respective time ranges, where # *p* < 0.0005; ^$^ *p* < 0.0005; ^ *p* < 0.0001; ^@^ *p* < 0.005.

We have, thus far, shown that VSV grown in HeLa cells harbors both CD46 and CD55, with the relative level of CD55 protein being much higher than that of CD46. HeLa cells are known to express higher levels of CD55 than CD46 [35], while A549 cells have higher levels of CD46 compared to that of CD55 [36]. The immunoblotting of equal concentrations of both the lysates confirmed these differential levels (Figure 6A). In order to compare the effects of differential levels of RCAs in two different cell lines in conferring protection to complement, VSV was grown in HeLa and A549 cells. They were harvested at time ranges of 0–6, 6–12, 12–18 and 18–24 h. Approximately 3 × 10^8^ pfu of virus was incubated either with PBS or normal human serum (NHS) for 30 min at 37 °C, and the infectivity of the remaining virus was titrated on monolayers of Vero cells (Figure 6B). It was observed that VSV grown in HeLa cells showed greater resistance to complement compared to the A549-grown viruses at all of the time ranges tested. While the VSV-HeLa (0–6 h) treated with NHS showed a 13.7-fold reduction in infectivity compared to the PBS treated sample, the VSV-A549 of the same time range showed a 57-fold reduction in plaque numbers (Table 3). Similarly, for the 12–18 h samples, the HeLa-grown VSV treated with NHS, when compared against the PBS controls, showed an 80-fold reduction in infectivity, whereas the infectivity in the corresponding A549-grown VSV was reduced by 2800-fold. The reduction in titers between the PBS- and NHS-treated samples in the 6–12 and 12–18 h time range was found to be significant in both the A549- and HeLa-grown viruses (*p* < 0.0005). Similarly, the differences in virus neutralization in the 6–12 and 12–18 h samples between the HeLa and A549 grown viruses were highly significant, with the HeLa-grown viruses being more resistant to NHS than the A549- grown VSV.

## 4. Discussion

The complement system plays a pivotal role as a first line of defense against invading pathogens, including viruses, and also contributes enormously in stimulating the adaptive immune responses. The importance of the role played by this system is apparent from the strategies that the viruses of several families have adopted over time in subverting the deleterious effects of the complement system. Vesicular stomatitis virus, a potent animal pathogen which causes acute infections in a wide range of mammalian hosts, is known to interact with the complement system. The exposure of VSV to human serum results in its neutralization by the combined action of natural IgM and complement [9]. It mostly activates the classical pathway, resulting in the deposition of C3b on the surface of the virus, thereby hampering its active attachment to susceptible cells [6]. In the fluid phase, activation of complement by VSV results in the rapid lysis of virus particles that are deficient in the RCAs, CD55 and CD46 [11]. The incorporation of biologically-active host RCAs with their corresponding biological activities intact has been well documented in several RNA viruses, including VSV [11]. The presence of host RCAs had the profound effect of delaying the virus neutralization by complement, thus offering a survival advantage to VSV, with CD55 having a dominant role over CD46. The protective role of the RCAs was elegantly demonstrated by culturing VSV in CHO cells overexpressing the RCAs [11]. However one pressing question that required addressing was the effect of VSV infection on the overall rates of the cellular expression of CD55 and CD46, and their pattern of recruitment by VSV.

Vesicular stomatitis virus is known to suppress host gene transcription and translation during infection [27,28,37,38]. Our results demonstrate that the CD55 protein levels were sustained at all of the time points tested, both in the whole cell lysates and on the cell surface. In sharp contrast, a marked decline in the expression of CD46 was observed on the cell surface and in the infected whole cell lysates. Further, the decline in CD46 mRNA as demonstrated by RT-PCR corresponded with the decline in protein levels. In the case of CD55, a significant drop in mRNA levels was observed but with a slight delay in comparison to CD46. The sustained levels of CD55 in the VSV-infected cells could otherwise be attributed to the specific up-regulation of CD55 by the virus. For example, certain paramyxoviruses, including PIV5 [39] and hRSV [40], have been shown to up-regulate membrane-associated RCAs CD55 and CD59, thus facilitating the enhanced incorporation of these proteins into the virus envelope, which is in striking contrast to the effect of VSV. Thus, the continued presence of CD55 in VSV-infected HeLa cells is not due to up-regulation, but rather the delay in the shutdown of CD55 transcription compared to CD46.

The differential levels of CD55 and CD46 in VSV-infected cells prompted us to investigate the pattern of incorporation of these proteins into the virus envelope as a function of time post infection. The 24 h window of the virus life cycle was broken down into four segments, with 0–6 h and 18–24 h representing early and late times, respectively, and the 6–12 h and 12–18 h representing intermediate times. The 0–6 h samples couldn’t be quantified due to the low levels of virus output. The immunoblotting of the remaining purified virus samples showed that the pattern of incorporation of both the RCAs was similar across the time intervals. The ratio of CD55 and CD46 to VSV proteins (G and M) remained the same, irrespective of the time of virus egress (Table 2). This raises the question of why the levels in the virus envelope did not change despite the changes in the levels of surface expression in infected cells. One possibility is that the levels of CD55 and CD46 remain constant in the membrane microdomains from which the virus buds, despite the overall decline in surface expression. With VSV, it is known that the virus buds from microdomains of varying sizes, from 100 to 400 nm [41]. In an infected cell, dynamic alterations in the membrane topology can result in the rearrangement and redistribution of membrane proteins, which in turn can affect the host protein incorporation into the virus envelope. Recent studies on enveloped viruses such as HIV, Ebola virus, and influenza virus show that these viruses bud from specific membrane microdomains enriched in cholesterol and sphingolipids called the ‘lipid rafts’ [42,43,44,45]. It is quite possible that, during VSV infection, the reorganization or redistribution of membrane-associated RCAs could occur, resulting in the concentration of these proteins in specific virus budding microdomains which are actively incorporated by the virus across all of the time ranges. The earlier electron microscopy analysis of VSV particles grown in CD55-CD46 double-positive CHO cells showed the enrichment of CD55 and CD46 in distinct regions of the virus envelope, further substantiating the microdomain hypothesis [11]. While CD46 is a trans-membrane protein, CD55 is a GPI-linked protein mostly enriched in lipid rafts, which could facilitate the efficient incorporation and enrichment in the virions. In the case of HIV, it was found that the disruption of lipid rafts resulted in decreased levels of RCAs in the virion [46]. VSV budding does not occur from lipid rafts [41]. However, the virus envelope is enriched in cholesterol and sphingolipids compared to the host plasma membrane from which it buds [47,48]. Thus, the membrane microdomains from which VSV buds resemble lipid rafts. This likely accounts for the incorporation of CD55 into the virus envelope.

The formation of convertases is a critical component in the progression of the complement cascade. With the half-life of classical pathway C3 convertase being 5 min at 37 °C [49], a regulator should be highly efficient to rapidly dissociate the convertase, which signifies the importance of CD55. Earlier studies and the current study (data not shown) have demonstrated that both the CD46 and CD55 associated with VSV are functional in nature, and can act as cofactors for C3b inactivation, or can accelerate the decay of C3 convertases [11]. Although a direct comparison cannot be drawn between the efficacy of cofactor versus decay accelerating activity, it may be speculated that the potency of CD55 is much higher than that of CD46. Contrary to the earlier discussions on the importance of CD55 over CD46, it could very well be argued that both CD46 and CD55 are equally important. In order to address this argument, VSV was cultured in HeLa cells which had high CD55 and low CD46, or A549 cells which harbor more CD46 than CD55, and their relative resistance to complement was assayed. VSV grown in A549 cells was found to be less resistant to complement than VSV grown in HeLa cells. This difference is quite evident in the neutralization data at the 6–12 h time point (Figure 6B). Thus, it could be confidently concluded that the relative significance of CD55 is more than that of CD46 in limiting the neutralizing effect of complement on VSV.

In summary, our data suggests that the criteria with which the virus selects a particular RCA may be a combination of factors which includes the nature of association of the protein with the lipid bilayer, and the overall distribution, concentration, and location of the RCA in the infected cells during the virus assembly and budding. It is quite possible that VSV preferentially buds off from microdomains enriched with CD55. Among the two RCAs, CD55 was found to confer greater protection to VSV against complement. These observations gain significance because VSV is currently exploited for its potential to be developed into oncolytic and vaccine vectors. These applications require the attenuation of VSV, and an attenuated vector harboring RCAs can have a significant advantage in overcoming complement and reaching the target. An attenuated vector, although safe for the host, is also exposed to both the innate and adaptive arms of the immune system. Shielding the vector with RCAs will provide the initial impetus required to bypass complement and thus reach the desired site. A baculovirus vector expressing the *Plasmodium falciparum* circumsporozoite protein, when shielded with CD55, had a protective efficiency of 60% compared to the vector without CD55 [50]. The generation of oncolytic or vaccine vectors in RCA-enriched cells can significantly protect the vectors from complement, as was elegantly reported in the case of a lentiviral vector pseudotyped with VSV-G and baculovirus gp64 [51]. Thus, it is highly important to take complement regulation into context for the rational design of vaccines and oncolytic vectors. Besides the application potentials, this study also provides valuable input into the phenomenon of recruitment of host proteins by VSV.

## Figures and Tables

**Table 1:** Gene identity, NCBI accession number, and primer sequences of the primers used for the real time PCR.

Gene	Assay ID	Accession No.	Amplicon Size (bp)
CD55	Hs00892618_m1	1604	98
CD46	Hs00611257_m1	4179	148
18s rRNA	Hs99999901_s1	HSRRN18S	187

**Table 2:** Levels of CD55 and CD46 associated with sucrose gradient-purified VSV from HeLa cells at various time intervals. ^a,b^ The concentration of the virion-associated CD55/CD46 was calculated from the pixel intensity of the known concentrations of rCD46 and rCD55 run in parallel (Figure 5A). The concentration of CD55 and CD46 is depicted as ng/5μg of VSV. ^c,f^ Based on the densitometric analysis of VSV-G or M, further normalization was performed relative to the values obtained at 6–12 h (taken as 1). The values indicate the decline in the concentration of G or M relative to 6–12 h. ^d,g^ The ratio of CD55 to VSV-G or VSV-M was calculated by dividing the CD55 concentration at a specific time point by the normalized levels of G or M. The values in ^d^ were obtained by dividing ^a^ by ^c^, and those in ^e^ by dividing ^b^ by ^c^. Similarly, the ratios in ^g^ and ^h^ were obtained by dividing ^a^ by ^f^ and ^b^ by ^f^, respectively.

Time Range of Harvest (h)	CD55 (ng/5 μg Virus) ^a^	CD46 (ng/5 μg Virus) ^b^	Normalized VSV-G Levels against the 6–12 h Levels ^c^	Ratio of CD55/G ^d^	Ratio of CD46/G ^e^	Normalized VSV-M Levels against the 6–12 h Levels ^f^	Ratio of CD55/M ^g^	Ratio of CD46/M ^h^
6-12	50	2.14	1	50	2.14	1	50	2.14
12-18	37.45	1.74	0.76	49.3	2.29	0.78	48.0	2.23
18-24	17.4	1.29	0.43	40.5	3.0	0.37	47.0	3.49

**Table 3:** Comparison of the neutralization of VSV grown in two different cell lines that express varying levels of RCAs.

Serial Number	Time Range of Harvest (h)	VSV-HeLa Fold Reduction (VSV+PBS/VSV+NHS)	VSV-A549 Fold Reduction (VSV+PBS/VSV+NHS)
1.	0–6	13.75	56.8
2.	6–12	10	236.36
3.	12–18	80	2823.52
4.	18–24	76.19	450

## Data Availability

Not applicable.
